# Acute effects of two different work-to-rest ratio of high-intensity interval training on brain-derived neurotrophic factor in untrained young men

**DOI:** 10.3389/fphys.2022.988773

**Published:** 2022-09-07

**Authors:** Kegang Zhao, Zhongyi Hu, Tao Wang, Lei Tian, Maoye Wang, Ruijiang Liu, Chongwen Zuo, Wang Jihua

**Affiliations:** ^1^ School of Physical Education of Shandong Normal University, Jinan, China; ^2^ Qilu Normal University, Jinan, China; ^3^ School of Physical Education of Liaocheng University, Jinan, China; ^4^ Capital Institute of Physical Education and Sports, Beijing, China; ^5^ Department of Information Science and Engineering of Shandong Normal University, Jinan, China

**Keywords:** high-intensity interval training, acute exercise, BDNF, neurotrophy, brain

## Abstract

**Background:** Aerobic exercise could produce a positive effect on the brain by releasing brain-derived neurotrophic factor (BDNF). In untrained healthy humans there seems to be a linear correlation between exercise duration and the positive effect of acute aerobic exercise on brain-derived neurotrophic factor levels. Therefore, we performed two different duration of high-intensity interval training protocols (HIIT), both known to improve cardiovascular fitness, to determine whether then have a similar efficacy in affecting brain-derived neurotrophic factor levels.

**Methods:** 12 untrained young males (aged 23.7 ± 1.8 years), participated in a randomized controlled cross-over trial. They underwent two different work-to-rest ratio high-intensity interval training protocols: high-intensity interval training 1 (30 min, 15 intervals of 1 min efforts at 85%–90% VO2max with 1 min of active recovery at 50%–60% VO2max) and HIIT2 (30 min, 10 intervals of 2 min efforts at 85%–90% VO2max with 1 min of active recovery at 50%–60% VO2max). Serum cortisol, brain-derived neurotrophic factor were collected at baseline, immediately following intervention, and 30 min into recovery for measurements using a Sandwich ELISA method, blood lactate was measured by using a portable lactate analyzer.

**Results:** Our results showed that the similar serum brain-derived neurotrophic factor change in both high-intensity interval training protocols, with maximal serum brain-derived neurotrophic factor levels being reached toward the end of intervention. There was no significant change in serum brain-derived neurotrophic factor from baseline after 30 min recovery. We then showed that both high-intensity interval training protocols significantly increase blood lactate and serum cortisol compared with baseline value (high-intensity interval training *p* < 0.01; high-intensity interval training 2 *p* < 0.01), with high-intensity interval training 2 reaching higher blood lactate levels than high-intensity interval training 1 (*p* = 0.027), but no difference was observed in serum cortisol between both protocols. Moreover, changes in serum brain-derived neurotrophic factor did corelate with change in blood lactate (high-intensity interval training 1 *r* = 0.577, *p* < 0.05; high-intensity interval training 2 *r* = 0.635, *p* < 0.05), but did not correlate with the change in serum cortisol.

**Conclusions:** brain-derived neurotrophic factor levels in untrained young men are significantly increased in response to different work-to-rest ratio of high-intensity interval training protocols, and the magnitude of increase is exercise duration independent. Moreover, the higher blood lactate did not raise circulating brain-derived neurotrophic factor. Therefore, given that prolonged exercise causes higher levels of cortisol. We suggest that the 1:1work-to-rest ratio of high-intensity interval training protocol might represent a preferred intervention for promoting brain health.

## 1 Introduction

### 1.1 Background

It is obvious that aerobic exercise improves cardiovascular fitness (Astorino et al., 2012; Ben-Zeev and Okun, 2021). Moreover, there is considerable evidence shows that aerobic exercise can lead to a positive impact on brain health by activating specific processes that promote neuronal differentiation and survival (Cotman and Engesser-Cesar, 2002; Vaynman et al., 2004b; Vaynman and Gomez-Pinilla, 2006).

One of the main mechanisms by which aerobic exercise promotes nervous system and brain health by releasing the expression of brain-derived neurotrophic factor (BDNF), an endogenous substance that play a major role in the brain (Oliff et al., 1998). BDNF is both a natural protein and a neurotrophy that mediate key functions of the central nervous system, such as neuroprotection, neuronal differentiation, neuronal protection and cell survival (Cotman and Engesser-Cesar, 2002; Lipsky and Marini, 2007). Aerobic studies with rodents models have been shown that BDNF mRNA expression and protein levels in hippocampal, striatum, and various cortical regions was elevated (Neeper et al., 1996; Widenfalk et al., 1999; Vaynman et al., 2004a; Rasmussen et al., 2009), even though acute exercise is performed (Huang et al., 2006; Ferris et al., 2007; Martínez-Díaz et al., 2020). It is noteworthy that the increased BDNF in the brain is positively correlated with the improvement of cognitive function, especially in learning and working memory and task switching that are hippocampus-dependent (e.g., Morris water maze task and the object recognition task) (Vaynman et al., 2004b; O’Callaghan et al., 2007).

Also, humans studies have reported that a single bout of aerobic exercise, also known as acute exercise increases BDNF levels in the brain, which provide a strong evidence for improving cognitive functions (Roig et al., 2013; Inoue et al., 2020; Martínez-Díaz et al., 2020; Tsai et al., 2021). However, this conclusion is obtained either measuring BDNF levels in the blood or from cohort data that based on their BDNF genotypes (Ozan et al., 2010). Modern hypothesis sometimes suggests that BDNF is mainly produced in the brain, and some of which could cross the blood-brain barrier (Pan et al., 1998) and flow to the periphery where it could be measured in serum and plasma (Lommatzsch et al., 2005). In addition, several studies show that BDNF can be produced by muscle satellite cells in the case of injury (Clow and Jasmin, 2010; Yu et al., 2017). From this perspective, the BDNF seems to originate from the muscle cells (Rasmussen et al., 2009), implying that BDNF produced by muscle cells cannot be response for the BDNF levels measured in serum or plasma (Saucedo Marquez et al., 2015).

Indeed, previous studies have reported that acute high-intensity interval training (HIIT) in-creased both serum and plasma BDNF in healthy individuals, also the positive effect of exercise on BDNF levels seems to be decided by training intensity and duration. HIIT protocol is an exhaustive training modality, interspersed with recovery periods. This type of exercise is characterized by time-saving efficiency, acceptability, compliance and safety, leading to better metabolic adaptations compared to other longer duration training modality. Normally, HIIT protocols performed at 90% of maximal work load with either a 1:1 work-to-rest ratio or a 2:1 work-to-rest ratio that is commonly used in clinical setting. In fact, both protocols are equally effective in improving aerobic capacity and reducing the risk of cardiovascular disease (Blue, 2018; Chin et al., 2020). For example, a recent study has proved that HIIT evokes larger serum BDNF levels compared with intense continuous exercise during similar exercise duration. (Saucedo Marquez et al., 2015). In that study, the blood lactate seemed to promote BDNF synthesis in the brain, and leading to higher serum BDNF levels (El Hayek et al., 2019; Hu et al., 2021). A single bout of HIIT elicits similar metabolic demands to other continuous exhaustive exercise modalities such as high-intensity functional training (HIFT), high-intensity continue training (FICT), high-intensity endurance training (FIET) and grade exercise test (GXT) (Murawska-Ciałowicz et al., 2021). For example, blood lactate levels >6 mmol/L have been reported following completion of different form of HIIT in healthy individuals (Saucedo Marquez et al., 2015; Rodriguez et al., 2018; García-Suárez et al., 2020). In addition to blood lactate, stress hormones (e.g., cortisol) are also increased after high-intensity protocols (Rodriguez et al., 2018; Martínez-Díaz et al., 2020). Given the variety of combinations of duration and interval time of HIIT, we have to consider which combination of training might be optimal for influencing the synthesis of BDNF. However, there is no study to compare whether HIIT protocols with different work-to-rest ratio and same intensity are equally effective in improving BDNF levels and may promote brain health to a similar extent.

HIIT protocol is a high-lactate producing exercise used to determine an individual’s aerobic capacity (e.g., VO2max, pVO2 peak). Besides, some studies have shown that this type exercise immediately increases serum BDNF levels, a response associated with synthesized lactate (Ferris et al., 2007; El Hayek et al., 2019). However, there is a lack of study, especially study in investigating the acute effects of HIIT with different work-to-rest ratio on serum BDNF levels. Here we investigated the change of serum BDNF levels by measuring it before, post immediately and after 30 min when participants performed either a 1:1 and a 2:1 work-to-rest ratio of HIIT protocol. Given that these two HIIT protocol exercise might both considerably increase blood lactate, and the blood lactate accumulation with the longer exercise duration. We hypothesized that a 2:1 work-to-rest ratio of acute protocol would elevate higher serum BDNF levels in untrained young men. Furthermore, we hypothesized that the change in lactate would directly correlate with the change in BDNF. Thus, the purpose of this study was to compare the acute effects of two different work-to-rest ratios of HIIT protocols on serum BDNF. However, opposite the blood lactate, the serum cortisol reduces BDNF synthesis. Thus, the second purpose of this study was to assess whether two different work-to-rest ratios of HIIT protocols increase the serum cortisol levels and suppress the BDNF response to exercise.

## 2 Materials and methods

### 2.1 Participants

**FIGURE 1:**
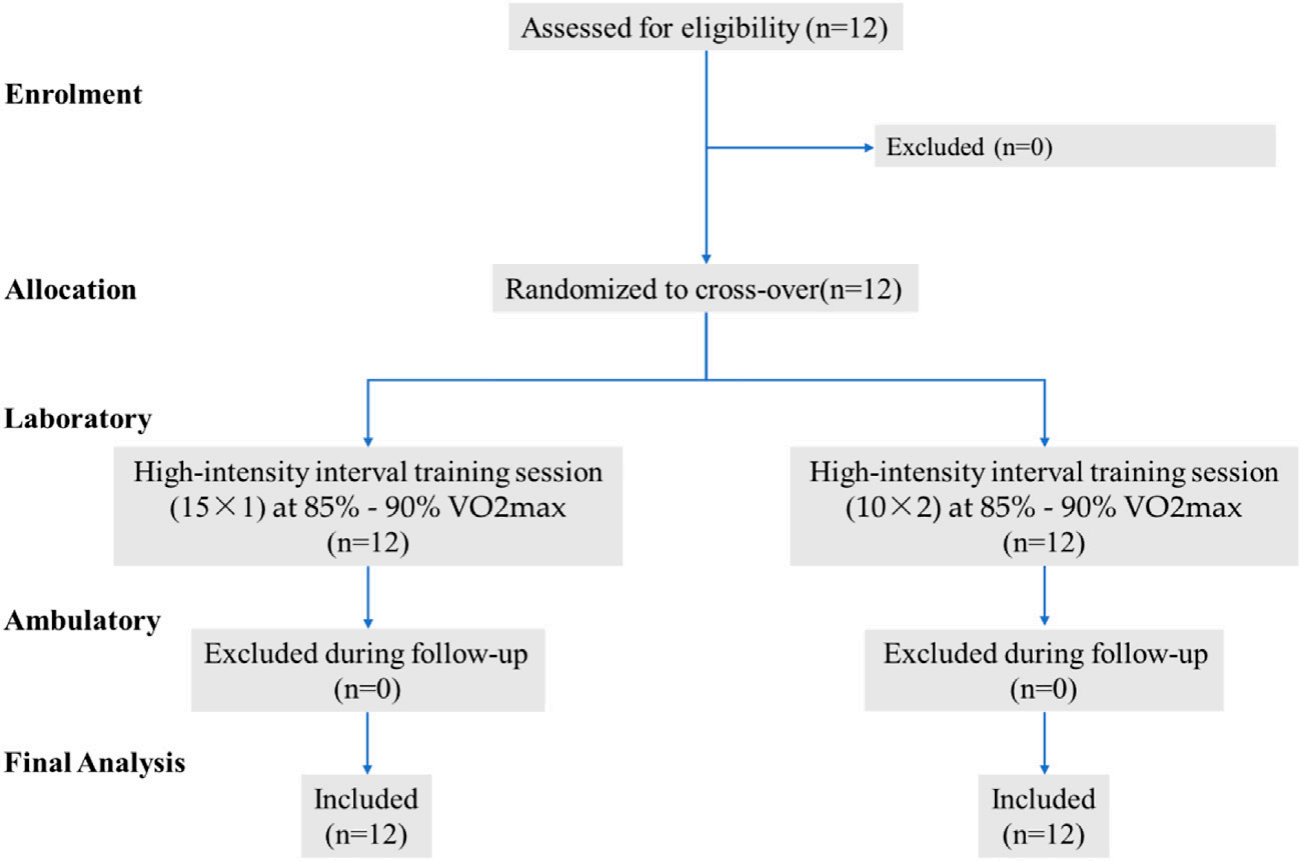
Sample flow chart.

Twelve untrained young male non-sports major college students (Mean ± SD, age: 23.7 ± 1.8 years; height: 1.77 ± 0.1 m; weight: 72.8 ± 5.8 kg; body mass index, BMI: 23.3 ± 1.1 kg/m^2^) volunteered to participate in this study (see Figure 1). The inclusion criteria for the selection of participants were as follows: 1) age between 18 and 30 years; 2) had not underwent any types aerobic training for at least 6 months; 3) no regularly smoke, drink alcohol, and other drug use (e.g., Donepezil); 4) good general health condition without overt chronic diseases (e.g., any cardiovascular, arthritis or metabolic disease) to interfere with this study. All participants were instructed not to attend any extra training and maintain normal eating habit throughout the study period. The ethical approval was granted by the Shandong Normal University ethical committee. Prior to study initiation, all the participants were informed of the risks and requirements of the training program, and voluntary consent was obtained from all of them. In order to minimize confounding effects, they were also informed to have an overnight fasting and not to consume alcohol or caffein, and perform any types of strenuous exercise 24 h prior to testing. Because any exercise may affect the physiological results after acute training.

The sample size was calculated based on a similar protocol from previous experimental design (Cabral-Santos et al., 2016). Moreover, with an effect size *f* ^2^ = 0.30, a power of 0.80, and a significance level of 0.05 (Cohen, 1992), the minimum sample size of 10 per group was found to be adequate using repeated measurements analysis of variance (ANOVA, G*Power 3.1; Heinrich Heine, Dusseldorf, Germany).

### 2.2 Experimental design

A cross-over controlled trial design was used in this study which involved two separate testing phases. During the first phase, all participants body composition was measured using bioelectrical impedance (TANITA BC-418MA) and an increasing progressively exercise test on a cycle ergometer (Ergoline 100 k, Germany) was performed in the first phase. The second phase included 1) a 10 min sitting rest; 2) pre-intervention measurement: a 5-ml venous blood withdrawal from antecubital vein; 3) participants were instructed to perform two different experimental conditions (HIIT1 intervention consisted of 15 x 1-min bouts of cycling on the ergometer and HIIT2 consisted of 10 x 2-min bouts of cycling on the same ergometer with 1 min of passive rest) in random order, at least 7 days is required between two interventions to avoid cross affect (Mendes et al., 2019); 4) post-intervention and 30-min post-assessment using the same procedure and order of measurements used for pre-measurement. All participants were informed of inhibiting vigorous activity during the previous 24 h and avoid food, caffeine and alcohol 12 h before measurement. In addition, all testing was finished at 8:00 a.m. to 11:00 a.m. of day in summer to control for circadian influence.

### 2.3 Maximal oxygen uptake test

Immediately following the body composition measurement, all participants performed a graded maximal exercise test to determine maximal oxygen uptake (VO2max) on a cycle ergometer. After a warm-up period of 5 min at 60 W, the workload was increased by 25 W/min until participants were volitional exhaustion (a constant speed was set at around 70 rpm). During the test procedure, heart rate (HR) was recorded beat-to-beat using a polar RS800 heart rate monitor (Polar, Kempele, Finland) to measure directedly the HR peak reached. The test was stopped when participants could not maintain a pedaling rate around 70 rpm/min. Peak work capacity (pVO2 peak) was measured and VO2max estimated as the highest 20 s mean value attained before exhaustion (Hawley and Noakes, 1992).

### 2.4 Experimental protocols

#### 2.4.1 High-intensity interval training protocols

All participants were informed to perform two different work-to-rest ratios of high-intensity interval training protocols, and a washout period of 1 week between the two training sessions was used to avoid cross effects. For the first training session, after an initial sitting rest period of 10 min and pre-intervention measurement, participants mounted the cycle ergometer. HIIT1 protocol started with a warm-up of 5 min at 60 W (a constant speed was set at around 70 rpm), followed by 15 bouts of 1 min (15 × 1) efforts at 85%–90% VO2max with 1 min of active recovery at 50%–60% VO2max between each high-intensity interval for a total of 35 min (Hood et al., 2011). After 1 week, participants performed HIIT2 protocol, which consisted of 10 bouts of 2 min (10 × 2) at 85%–90% VO2max with similar active recovery for a total of 35 min. Two training sessions for the participants were directly supervised by the lab administrator. During both training sessions, standardized verbal stimuli were provided.

#### 2.4.2 High-intensity interval training heart rate and ratings of perceived exertion assessment

During both training period, each participant’s HR was monitored continuously by an HR monitor (PolarRS800, Kempele, Finland). Mean and maximal HR were recorded for each bout of training. The Borg’s 0–20 scale was shown to participants at the beginning and end of each bout of training to measure their ratings of perceived exertion (RPE) during throughout the training period.

#### 2.4.3 Blood sampling and analysis

A 5 ml blood samples withdrawal via an aseptic technique from the antecubital vein were drawn before, post, and 30-min post HIIT protocol for the determination of blood lactate, cortisol and serum BDNF levels. The blood samples were allowed to clot (BD Vacutainer Plus SST) at room temperature and were then centrifuged at 3,000 rpm for 15 min at 4 C. The supernatant was decanted and kept in a–80°C freezer until further analysis of the cortisol and BDNF levels. Blood lactate was assessed with a portable lactate analyzer (EKF, Lactate Scout 4, Germany) that was calibrated to the manufactory’s instructions before each test. The levels of serum cortisol and BDNF were analyzed by the enzyme-linked immunosorbent assay (ELISA) technique using the human free kit purchased from Shanghai Xinyu Bioengineering Institute (XY-0030c and SEA011Hu; Shanghai, China), with a sensitivity range from 12.5 to 800 ng/ml and 156 pg/ml to 10,000 pg/ml, and the intra-assay and inter-assay variations were<10% and <10% for cortisol and <10% and <12% for BDNF, respectively.

#### 2.4.4 Statistical analysis

All Statistical analyses were performed using SPSS version 24.0 Windows (SPSS, Inc., Chicago, IL, United States). The significance level was considered as *p*-value of <0.05, and all data were presented as mean ± standard deviation (SD). Kolmogorov–Smirnov and Shapiro-Wilk’s W tests were used to confirm normal distribution of all variables. Two-way repeated measures analysis of variance (ANOVA) was applied to determine the training effects of within-group factors condition (HIIT1 and HIIT2), time (before, post and 30-min post), while a two-way ANOVA (condition× time) was used to verify differences for all variables between groups. Post-hoc tests were applied using the Bonferroni correction. In addition, effect size (ES) was calculated using the Eta-squared (η^2^)—value proposed by Cohen (Cohen, 1992); thus, the ES was classified as: “small” less than 0.01, “medium” from 0.01–0.138, “large” higher than 0.138. The correlation between the before to post changes in variables was examined with the Pearson product-moment correlation which was set at 0.500 for a positive correlation.

## 3 Results

### 3.1 Participants

Table 1 summarizes the main characteristics of the participants obtained throughout the graded maximal exercise test. Base on the mean age of participants (23.7 ± 1.8 years), the mean HRmax for exhaustion in the incremental graded exercise test was 175.2 ± 11.0 bpm. Considering the protocol performed, this heart rate was appropriate to define VO2max (45.3 ± 5.9 ml/kg/min), at which the participants produced a mean power of 298.8 ± 44.8 W.

**TABLE 1:** Demographic characteristic of participants.

Variables	Mean ± SD	Range (min to max)
Age (years)	23.7 ± 1.8	21–28
Height (m)	1.77 ± 0.1	1.64–1.88
Weight (kg)	72.8 ± 5.8	63.0–81.3
BMI (kg/m^2^)	23.3 ± 1.1	21.0–24.8
HRmax (bpm)	175.2 ± 11.0	160.0–197.0
PVO2 peak (w)	298.8 ± 44.8	209.0–388.4
VO2max (ml/kg/min)	45.3 ± 5.9	37.0–54.4

BMI, body mass index, VO2max maximal oxygen consumption, HRmax, maximal heart rate, pVO2 cycling power corresponding, SD, standard deviation.

### 3.2 Heart rate and ratings of perceived exertion response to high-intensity interval training

**FIGURE 2:**
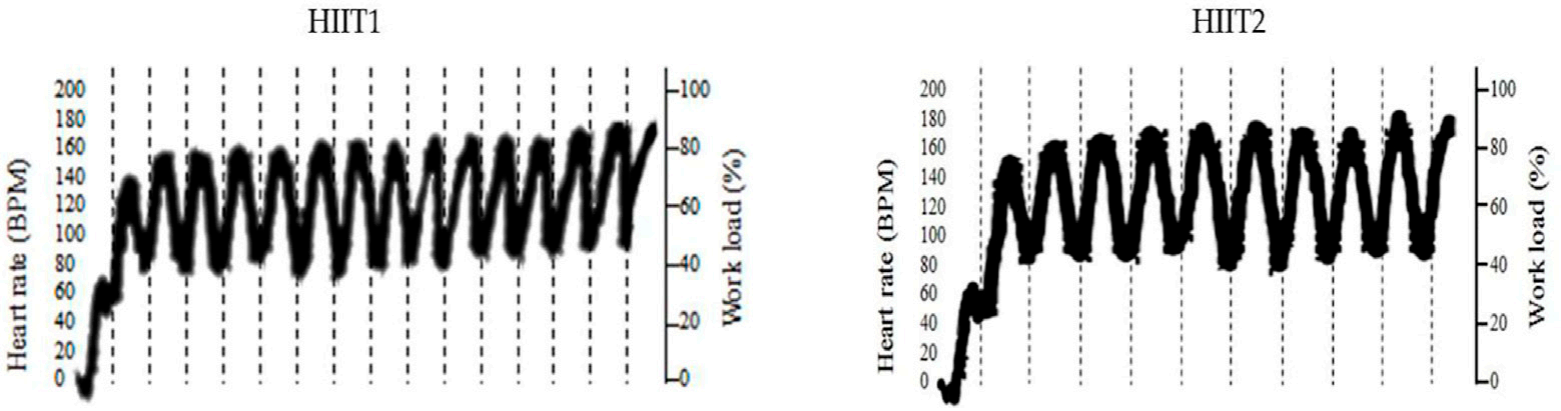
Heart rate monitor during two HIIT protocols period.

As can be seen in the Figure 2, HR values gradually increased from the first to the last repetition during the HIIT intervention period. Maximal HR showed a progressively growth trend from 139.0 ± 2.2 to 168.0 ± 2.8 bmp in HIIT1 group and 146.4 ± 1.9 to 176.5 ± 3.4 bmp in HIIT2 group; Regarding to RPE scale, it showed a similar tendency to that observed in HR. RPE scale score was gradually increased from 9.8 ± 1.5 to 18.8 ± 1.0 points in HIIT1 group and 10.8 ± 1.4 to 19.6 ± 0.5 points in HIIT group at the beginning to end.

### 3.3 Lactate, cortisol and brain-derived neurotrophic factor assessment

**FIGURE 3:**
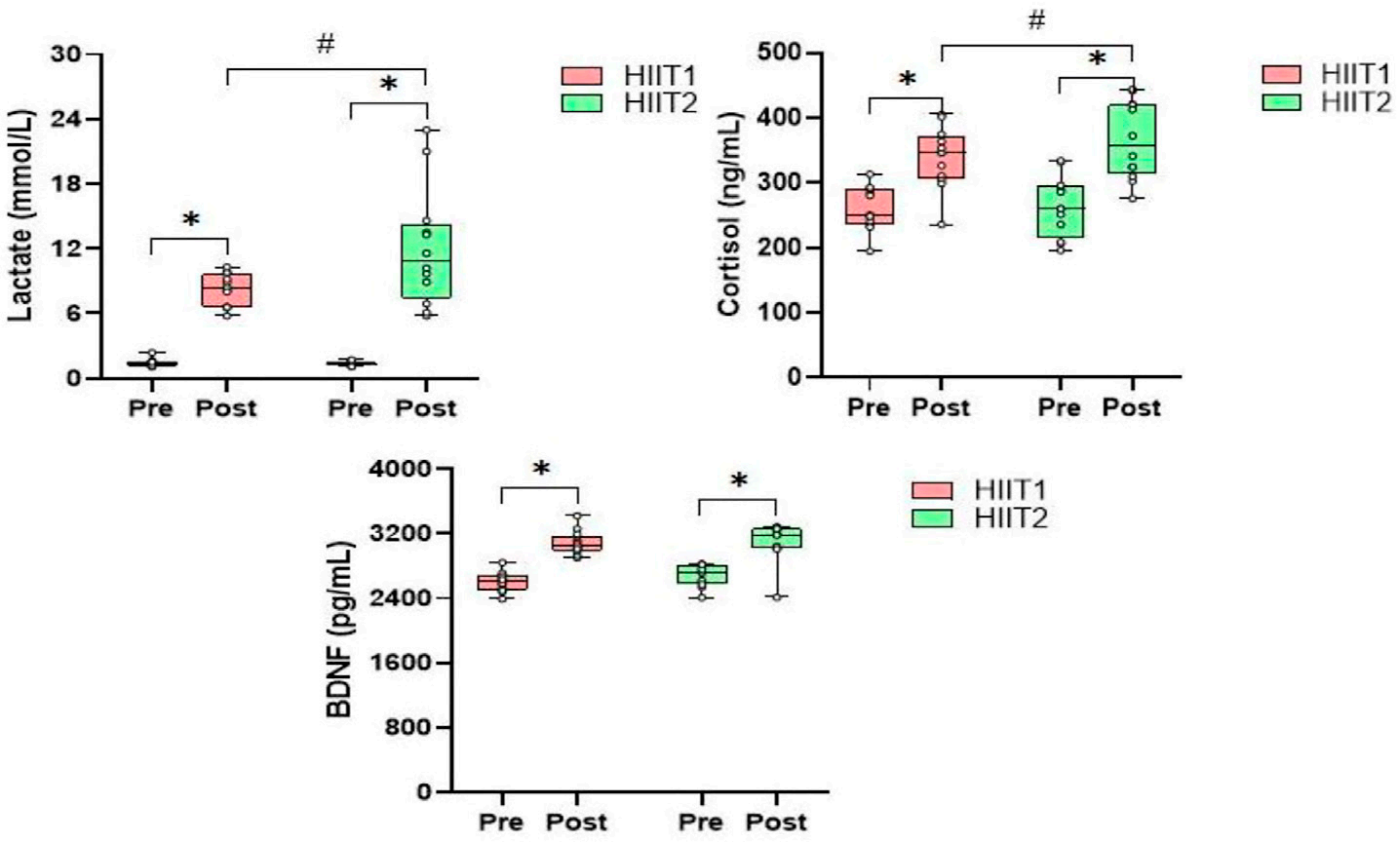
The relative changes (mean) in blood lactate, serum cortisol and BDNF levels during the HIIT1 and HIIT2 protocol Pre and Post. *p < 0.01 post vs. Pre, ^#^ *p* < 0.05 difference between groups.

Table 2 presents the mean values of blood lactate, serum cortisol and BDNF levels in the immunoassay before, post and 30-min post intervention for both HIIT1 and HIIT2 conditions. Across all measures, there was no difference between HIIT1 and HIIT2 on baseline levels for blood lactate, serum cortisol and BDNF; however, there was a significant effect of time (*p* < 0.01, η^2^ = 0.724) and a significant effect of condition (*p* < 0.01, η^2^ = 0.038), and a significant effect of condition × time interactions (*p* < 0.05, η^2^ = 0.032) on the blood lactate. The post-hoc analyses showed that the blood lactate levels post intervention increased significantly as compared to pre intervention, and the blood lactate levels were elicited to increase significantly via both the HIIT1 (Pre vs. Post: 1.4 ± 0.4 mmol/L vs. 8.4 ± 1.5 mmol/L) and HIIT2 (Pre vs. Post: 1.4 ± 0.2 mmol/L vs. 12.1 ± 5.5 mmol/L) intervention protocols. The 95% CIs suggested both HIIT protocols resulted in significant lactate changes (Table 3). Figure 3 shows the growth was greater in the HIIT2 group compared to HIIT1 group (*p* < 0.05). However, they decreased significantly over the next 30 min (*p* < 0.01), but did not return to the pre-exercise levels, which in HIIT2 group was still higher than HIIT1 group (*p* < 0.01).

**TABLE 2:** Blood lactate, serum cortisol and BDNF responses measured at the assessment time-points.

Variables	Group	Pre	Post	30-min post	Interaction	*p-*value	η^2^
Lactate (mmol/L)	HIIT1	1.4 ± 0.4	8.4 ± 1.5^a^	2.2 ± 0.4^b^ ^,^ ^c^	Time	0.001	0.724
	HIIT2	1.4 ± 0.2	12.1 ± 5.5^a^ ^,^ ^d^	3.7 ± 1.4^b^ ^,^ ^c^ ^,^ ^d^	Group	0.009	0.038
					Time×Group	0.017	0.032
Cortisol (ng/ml)	HIIT1	254.8 ± 31.7	328.4 ± 42.7^a^	269.6 ± 44.3^b^	Time	0.001	0.356
	HIIT2	267.6 ± 46.7	376.1 ± 56.4^a^	348.0 ± 78.4^c^ ^,^ ^d^	Group	0.034	0.138
					Time×Group	0.013	0.046
BDNF (pg/ml)	HIIT1	2,602.5 ± 121.1	3,081.7 ± 147.0^a^	2,576.7 ± 225.6^b^	Time	0.001	0.791
	HIIT2	2,682.8 ± 135.0	3,135.6 ± 143.4^a^	2,669.5 ± 144.8^b^	Group	0.164	0.022
					Time×Group	0.755	0.001

BDNF, brain-derived neurotrophic factor, Pre pre-exercise intervention, Post just at the end of exercise, 30-min Post 30 min after exercise, η^2^ eta square.

a *p* < 0.01 Post vs. Pre.

b *p* < 0.01 30-min post vs. Post.

c *p* < 0.05 30-min post vs. Pre.

d *p* < 0.05 difference between groups.

**TABLE 3:** Mean changes (95% confidence intervals, 95%CIs) from pre, post and 30-min post for Lactate, Cortisol and BDNF.

Variables		MD	95% CIs for MD		t	*p*-value
Lactate			Lower	Upper		
HIIT1 Pre	HIIT1, Post	−6.783	−9.719	−3.848	−7.179	0.001
	HIIT1, 30 min-Post	−0.808	−3.744	2.127	−0.855	1.000
HIIT1 Post	HIIT1, 30 min-Post	5.975	3.040	8.910	6.323	0.001
HIIT2 Pre	HIIT2, Post	−10.692	−13.627	−7.756	−11.315	0.001
	HIIT2, 30 min-Post	−2.292	−5.227	0.644	−2.425	0.293
HIIT2 Post	HIIT2, 30 min-Post	8.400	5.465	11.335	8.890	0.001
Cortisol						
HIIT1 Pre	HIIT1, Post	−73.584	−118.662	−28.505	−5.067	0.001
	HIIT1, 30 min-Post	−14.747	−59.825	30.332	−1.016	1.000
HIIT1 Post	HIIT1, 30 min-Post	58.837	13.759	103.916	4.052	0.003
HIIT2 Pre	HIIT2, Post	−108.523	−153.601	−63.444	−7.473	0.001
	HIIT2, 30 min-Post	−80.399	−125.477	−35.320	−5.537	0.001
HIIT2 Post	HIIT2, 30 min-Post	28.124	−16.954	73.203	1.937	0.888
BDNF						
HIIT1 Pre	HIIT1, Post	−479.167	−590.887	−367.447	−13.317	0.001
	HIIT1, 30 min-Post	25.833	−85.887	137.553	0.718	1.000
HIIT1 Post	HIIT1, 30 min-Post	505.000	393.280	616.720	14.035	0.001
HIIT2 Pre	HIIT2, Post	−452.833	−564.553	−341.113	−12.585	0.001
	HIIT2, 30 min-Post	13.250	−98.470	124.970	0.368	1.000
HIIT2 Post	HIIT2, 30 min-Post	466.083	354.363	577.803	12.953	0.001

BDNF, brain-derived neurotrophic factor, Pre pre-exercise intervention, Post just at the end of exercise, 30-min Post 30 min after exercise, 95% CIs, 95% confidence intervals, MD, mean difference.

Regarding the serum cortisol levels, there was a significant effect of time (*p* < 0.01, η^2^ = 0.356) and a significant effect of condition (*p* < 0.05, η^2^ = 0.138), and a significant effect of condition × time interactions (*p* < 0.05, η^2^ = 0.046) on the serum cortisol levels. The post-hoc analyses showed that both groups experienced a significant increase after HIIT intervention protocols (*p* < 0.05), but the serum cortisol levels returned to pre-exercise levels in the next 30-min only in HIIT1 group. In addition, post-hoc analyses also showed significant differences between HIIT1 and HIIT2 in the next 30 min (*p* < 0.05).

As showed in Table 2, there was a significant effect of time effects (*p* < 0.05, η^2^ = 791), whereas neither condition nor the condition × time interactions was significant for peripheral serum levels of BDNF. The post-hoc analyses revealed that the serum BDNF levels were found to increase significantly via HIIT1 (Pre vs. Post: 2,602.5 ± 121.1 ng/ml vs. 3,081.7 ± 147.0 ng/ml) and HIIT2 (Pre vs. Post:

2,682.8 ± 135.0 ng/ml vs. 3,135.6 ± 143.4 ng/ml) just after intervention (Figure 3) The 95% CIs suggested that both HIIT protocols led to a significant increase in serum BDNF. However, they returned to the pre-exercise levels in the next 30 min, with no significant differences between groups.

### 3.4 Correlation between variables

**FIGURE 4:**
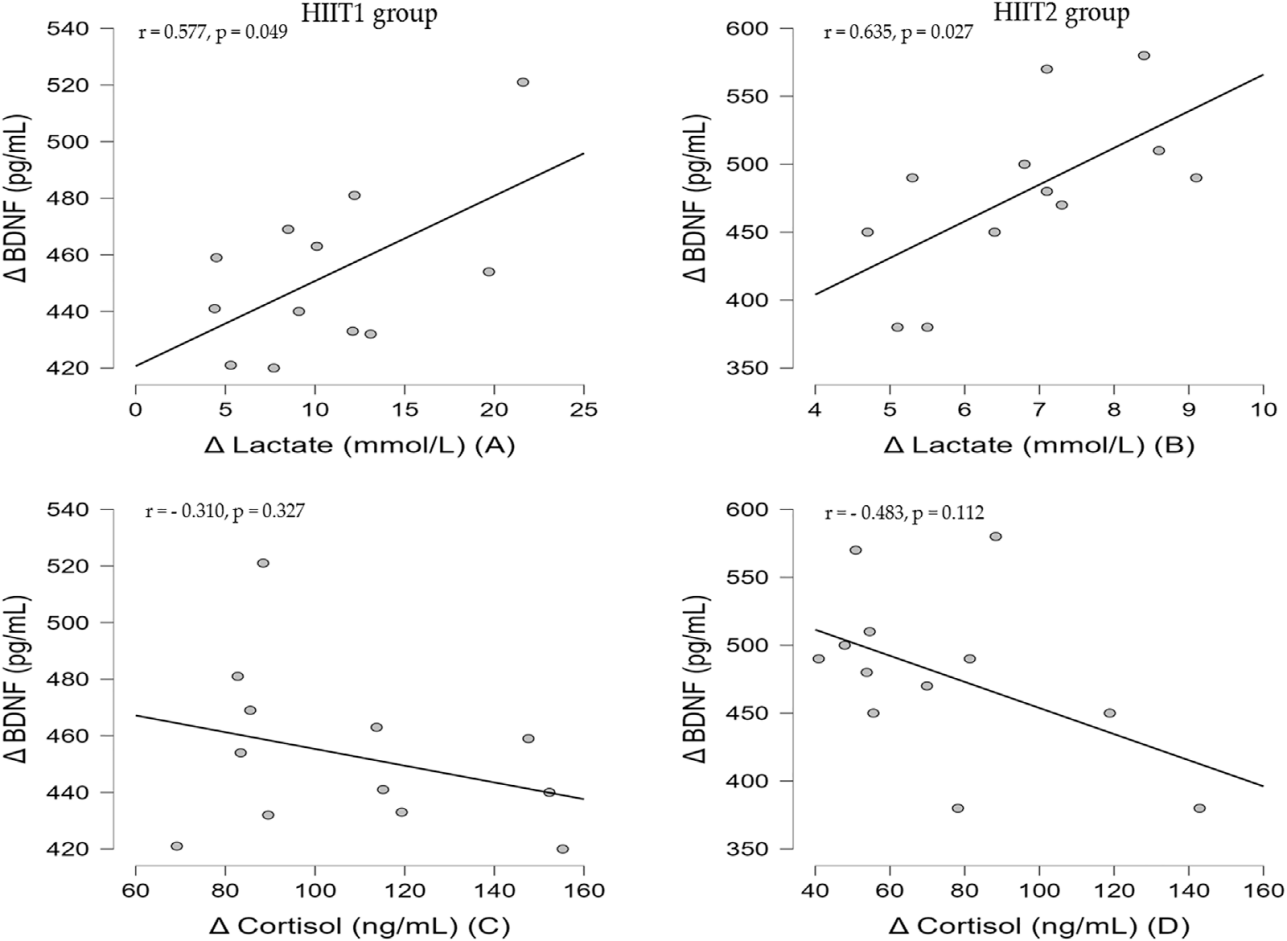
Correlation between delta value in blood lactate, serum cortisol and serum BDNF for HIIT1 group **(A** and **C)** and HIIT2 group **(B** and **D)**.

The Figure 4 revealed the Pearson correlation coefficients for the association between delta value in blood lactate, serum cortisol and BDNF. In both groups, there was a significant positive correlation between the delta vale in serum BDNF and the blood lactate (HIIT1 group: *r* = 0.577, *p* < 0.05, Figure 2A; HIIT2 group: *r* = 0.635, *p* < 0.05, Figure 2B). However, there was no significant correlation (*p* > 0.05) between the delta value in BDNF and cortisol for both groups.

## 4 Discussion

There appears to be growing interest in research on the link between exercise duration and neurocognitive molecular biomarkers. Therefore, in the present study, the acute effects of two different work-to-rest ratios of HIIT protocols on serum BDNF levels were assessed in untrained young men. Additionally, the correlation between changes in blood lactate, serum cortisol and serum BDNF levels induced by the two different exercise duration was inspected to comprehend the potential mechanism behind such changes. The present study findings verify that different work-to-rest ratio of HIIT protocols (1:1 and 2:1 work-to-rest ratio) could be an effective strategy to upregulate serum BDNF expression in untrained young men. In addition, both protocols resulted in significantly increase in blood lactate and serum cortisol levels compare to pre-intervention. Moreover, the increase in blood lactate was significantly higher for the HIIT2 protocol after exercise. However, there were no significant correlations between the changes in serum BDNF and the serum cortisol levels induced by the two acute HIIT protocols. A significant correlation was only observed in the changes in serum BDNF and blood lactate levels with two acute HIIT protocols.

We observed that serum BDNF levels increased significantly relative to pre-exercise levels whether participants performed 1 min for 15 bouts or 2 min for 10 bouts of HIIT interventions. Which is consistent with previous study in humans (Saucedo Marquez et al., 2015; Cabral-Santos et al., 2016; Martínez-Díaz et al., 2020; Tsai et al., 2021; Hendy et al., 2022). Although the training duration of the two HIIT protocols was different, with maximum levels of serum BDNF being observed toward the end of intervention. After 30 min recovery, the serum BDNF levels returned quickly to pre-exercise levels such that the 30 min post exercise measurement was not significantly different from levels at the beginning. These findings are very consistent with findings reported by Martínez-Díaz et al. (2020), who explained that BDNF levels increased after intervention, reaching highest levels after 10 bouts of cycling and returned to baseline values after 30 min of rest. Our data extend these previous findings because we determined exercise intensity based on the VO2max measurement, while Martínez-Díaz et al. (2020) used the mean value of VO2 peak in the highest 20 s as the exercise intensity. Obviously, their exercise intensity seems to be slightly higher than our training protocols. Moreover, our results show that serum BDNF change is similar for both HIIT protocols, and suggest that HIIT protocols (15 bouts of 1 min efforts vs. 10 bouts of 2 min efforts at 85%–90% VO2max with 1 min of active recovery) of at least 30 min are required to increase serum BDNF levels by 18.4% and 16.9%, respectively. As previous literature mentioned, the change in serum BDNF levels after performing high intense exercise vary from 11.7 to 410% in both healthy people and patients (Klein et al., 2011). Murawska-Ciałowicz et al. (2021) et al. recently suggested that using four different forms of high intensity training methods could increase the level of BDNF by 10–38%, especially the long-term impact of high intensity functional training and interval training on the levels of BDNF is more prominent. In another study it was also reported the lowest growth rate of serum BDNF that seems to influence behavior (Winter et al., 2007). They found that a 12% increase in serum BDNF led to a 20% increase in new words learning. Others evidence demonstrated that a 10–30% increase in serum BDNF can improve the Stroop task (Ferris et al., 2007) and Recognition task performance (Griffin et al., 2011). Therefore, the pre to post changes in serum BDNF observed in this study conformed to the range of values that have previously reported improvements in neurocognitive function.

Several studies have suggested that BDNF levels assessed in serum is reflective of BDNF expression in the brain tissue. For example, Klein et al. (2011) proved in the animal model that the BDNF levels in brain tissueares related to the peripheral levels of BDNF. Moreover, Sartorius et al. (2009) also demonstrated in the animal model of electroconvulsive therapy that a positive relationship between brain tissue and serum BDNF levels. Significantly, it has been confirmed that most of BDNF crossed the blood-brain barrier and measured in the periphery during exercise and rest (Rasmussen et al., 2009; Walsh and Tschakovsky, 2018). Hence, most researchers believe that it is reliable to measure the levels of circulating BDNF to reflect brain health.

In terms of the associations between serum BDNF responsivity to acute HIIT intervention and stress response, only a significant correlation between changes in blood lactate and serum BDNF levels were found, though the expectation of changes in the lactate, cortisol and BDNF levels induced by both HIIT protocols. It is well known that the BDNF is a protein sensitive to intense acute training (Saucedo Marquez et al., 2015; Cabral-Santos et al., 2016). Previous studies found that blood lactate may be an important regulatory molecule responsible for inducing BDNF synthesis in the brain (Rojas Vega et al., 2006; El Hayek et al., 2019; Müller et al., 2020). In fact, the blood lactate increased significantly after both HIIT interventions in this study, and the higher blood lactate level was observed following HIIT2 protocol. The difference in blood lactate levels between the two groups may could be due to the long exercise duration in HIIT2 group, which leads to long-term hypoxia in skeletal muscle and promotes the excessive accumulation of lactate. On the other hand, the recovery of muscle oxygen content in skeletal muscle is in a curve form, so the frequent intervals may be more conducive to the recovery of muscle oxygen in skeletal muscle, and the recovery of muscle oxygen and the elimination of blood lactate complement each other. The rapid recovery of muscle oxygen and the high oxygen content in muscle tissue can promote the oxidative clearance of lactate after exercise (Buchheit and Laursen, 2013). In this study, the number of intervals time in HIIT1 group is more than that in HIIT2 group (15 intervals vs. 10 intervals), which means that skeletal muscle has more time to promote muscle oxygen recovery, so that lactate could quickly recover to the pre intervention level after exercise.

To the best of our knowledge, we are the first to report an exercise duration – dependent induction of serum BDNF in untrained young men. The change in blood lactate did correlate with the change in serum BDNF in both HIIT protocols, and this findings was consistent with previous study (Gold et al., 2003; Ferris et al., 2007). Interestingly, Müller et al. (2020) revealed the potential mechanisms of lactate-BDNF interaction following exercise that the intensity and duration of exercise determine the level of lactate, which can cross the blood-brain barrier (BBB) via different monocarboxylate transporters (MCT’s). Also, lactate binding to the hydroxycarboxylic acid receptor (HCAR1) at the BBB can induce angiogenesis. In neurons, lactate plays variety of neurotrophic and metabolic roles through the direct combination of MCT transmembrane transport and HCAR1. Specifically, lactate can induce the PGC1α- FNDC5—BDNF pathway through SIRT1 activation to enhance BDNF expression and synthesis (Wrann et al., 2013). Additionally, a recent study also confirmed that HIIT induced peripheral lactate secretion is responsible for regulating mitochondrial quality control system for mitochondrial function and thus may contribute to BDNF expression (Hu et al., 2021). Whilst this could be a possible explanation for the correlation between lactate and BDNF in this study and others. In the current study, the blood lactate increased significantly after both training protocols, although the higher blood lactate levels were observed following HIIT2 protocols, there were similar BDNF levels following both training protocols. The current results do not support our hypothesis that peripheral lactate produced following longer duration of HIIT protocol (2:1 work-to-rest) would elicit higher circulating BDNF levels. Another study also observed similar findings (García-Suárez et al., 2020). The restricted secretion of BDNF can be partly explained by the increase of serum cortisol after high-intensity exercise (Issa et al., 2010). The cortisol of two groups reported in this study, could indirectly indicate the higher cortisol after HIIT2 protocol is an inhibitor of peripheral BDNF synthesis.

Cortisol is the human body’s primary stress hormone (Sapolsky et al., 2000). Several studies have been demonstrated that elevated cortisol levels inhibit neurogenesis and neuronal plasticity, which downregulate BDNF expression (Schaaf et al., 1998; Quesada et al., 2012; Naveen et al., 2016; Martínez-Díaz et al., 2020), it means a negative relationship between cortisol and BDNF(Smith et al., 1995); However, this correlation in response to exercise still remains unclear. In fact, in this study and other human studies (Goekint et al., 2008; Moreira et al., 2018), there was no evidence of such a simple linear relationship between serum cortisol and BDNF following both acute HIIT protocols. In addition, there was sufficient evidence to prove the relationship between cortisol response and exercise intensity. Rojas Vega et al. (2006)found no change in cortisol levels after 10 min moderate-intensityexercise. Whereas it significantly increases after 10 min of grade incremental exercise test. VanBruggen et al. (2011) further supported the results mentioned above that serum cortisol significantly increased only after high-intensity exercise. On the other hand, Tauler et al. (2014) reported that the magnitude of the increase in serum cortisol levels depends on the intensity and duration of exercise. In our results, both HIIT protocols with different exercise duration significantly increase serum cortisol levels post-intervention immediately, indicating that both HIIT protocols can effectively induce the human body’s stress response. Nonetheless, the change in cortisol between both groups did not reach significant difference and there was no correlation between the serum cortisol levels and BDNF for either HIIT protocol.

There are some limitations should be noted. First, because of small sample size, we only tested untrained young men in this study, who only represent a small part of the whole group, and it was difficult to generalize our findings to other groups such as athletes, women, elderly and patients. Consequently, our results should be interpreted with caution. Moreover, we did not set up a moderate-intensity continuous exercise group, which could have provided more specific information on how exercise regulates BDNF expression. Therefore, future study is required to determine the relationship between BDFN and stress hormones in a large sample under different exercise intensity.

## 5 Conclusion

In summary, the present findings showed that the stress induced by different wort-to-rest ratio of HIIT induces a remarkable response of serum cortisol, BDNF and blood lactate in untrained young men. We demonstrated that serum BDNF levels gradually increase during both HIIT protocols with different exercise duration, and this increase is temporary, with serum BDNF levels returning to pre-intervention levels 30 min after the end of training. Moreover, we detected a positive correlation between the change in serum BDNF and blood lactate among both HIIT protocols of different exercise duration, which might suggest that blood lactate accumulation caused by high-intensity exercise could coincide with activation of a signaling cascade that triggers the release of BDNF in the brain. Nevertheless, given that HIIT protocols with different exercise duration induce similar serum BDNF levels, and prolonged exercise causes higher levels of cortisol, we recommend 1:1 work-to-rest ratio of HIIT protocol as a potential intervention for improving BDNF levels in the brain, which might improve neural plasticity, cognitive and neurological function.

## Data Availability

The raw data supporting the conclusions of this article will be made available by the authors, without undue reservation.
